# Long Non-coding RNA Double Homeobox A Pseudogene 8: A Novel Oncogenic Propellant in Human Cancer

**DOI:** 10.3389/fcell.2021.709069

**Published:** 2021-09-23

**Authors:** Chen Xue, Xiaolu Cai, Junjun Jia

**Affiliations:** ^1^State Key Laboratory for Diagnosis and Treatment of Infectious Diseases, National Clinical Research Center for Infectious Diseases, Collaborative Innovation Center for Diagnosis and Treatment of Infectious Diseases, The First Affiliated Hospital, College of Medicine, Zhejiang University, Hangzhou, China; ^2^Department of Oncological Surgery, The First Affiliated Hospital, College of Medicine, Zhejiang University, Hangzhou, China; ^3^Division of Hepatobiliary and Pancreatic Surgery, Department of Surgery, The First Affiliated Hospital, College of Medicine, Zhejiang University, Hangzhou, China

**Keywords:** lncRNA, DUXAP8, cancer, molecular mechanism, tumorigenesis

## Abstract

A growing number of studies are reporting important roles played by long non-coding RNAs (lncRNAs) in various pathological and physiological processes. LncRNAs are implicated in numerous genomic regulatory functions at different levels, including regulation of transcription, post-transcriptional processes, genomic stability, and epigenetic genome modifications. Double homeobox A pseudogene 8 (DUXAP8), a novel lncRNA, has been reported to be involved in many cancers, including gastric, colorectal, esophageal, bladder, oral, ovarian, lung, and pancreatic cancers as well as hepatocellular carcinoma (HCC). DUXAP8 plays specific oncogenic roles *via* numerous malignancies promoting pathways. DUXAP8 is frequently dysregulated in multiple cancers, acting as a sponge to downregulate various tumor-suppressing microRNA activities. In this review, we comprehensively explore DUXAP8 expression and prognosis across cancer types, and systematically summarize current evidence concerning the functions and molecular mechanisms of DUXAP8 in tumorigenesis and progression. We conclude that DUXAP8 is a potential biomarker and therapeutic target for multiple cancers.

## Introduction

Cancers collectively represent a life-threatening disease with major impact on public health (the second leading cause of death worldwide). An estimated 18.1 million new cancer cases and 9.6 million deaths occurred in 2018 (Bray et al., 2018). The predicted number of new cancer patients is projected to be 14 million in 2035 (Pilleron et al., 2019).

The human genome project has revealed that there are approximately 20,000–25,000 protein-coding genes in the human genome that account for 2% of the total human genome sequence (Ponting et al., 2009). Genetic mutations associated with diseases are commonly located in non-coding regions of the human genome (Elkon and Agami, 2017; Darbellay and Necsulea, 2020). The majority of the human genome is not protein-coding, and other transcriptionally active regions were originally considered to be transcriptional noise. As such, they attracted little attention (Ponting et al., 2009; Evans et al., 2016; Yang et al., 2020). Recent studies have demonstrated that expression of the non-coding RNAs produced by these regions is systematically altered in cancers, and displays potential correlations with protein coding gene expression, demonstrating the importance of long non-coding RNAs (lncRNAs) in tumor formation, development, and progression (Goodall and Wickramasinghe, 2021; Statello et al., 2021). As lncRNAs are highly enriched in the genome, they are dynamically regulated in cell-, tissue-, and development-specific manners (Sun et al., 2018).

Double homeobox A pseudogene 8 (DUXAP8), according to the HUGO Gene Nomenclature Committee, is a newly identified lncRNA located on 22q11.1. DUXAP8 is approximately 2,307 bp long. Recent studies have reported that DUXAP8 mRNA is substantially upregulated in many cancer tissues, including pancreatic, bladder, colon, lung, ovarian, and breast cancers (Jiang et al., 2018; Lin et al., 2018; Chen et al., 2020a; He et al., 2020; Meng et al., 2020; Wang et al., 2020; Yang et al., 2021), as well as thyroid, hepatocellular and renal cell carcinomas (RCCs) (Hu et al., 2020; Wang et al., 2020; Pang and Yang, 2021), and glioma, compared to corresponding non-tumor tissues. Emerging literature supports that overexpressed lncRNA DUXAP8 might function as a sponge in cancer tissues, targeting tumor suppressive microRNAs, thereby facilitating target oncogene signaling pathway activity and promoting tumor development and progression.

In this review, we comprehensively summarize tissue and developmental stage-specific lncRNA DUXAP8 mRNA expression and systematically describe DUXAP8-associated regulatory mechanisms based on current literature.

### The Transcriptional Level of Double Homeobox A Pseudogene 8 Across Pancancer

To characterize mRNA expression levels of DUXAP8 in 33 different cancers, we developed gene expression profiling interactive analysis 2 (GEPIA2) and determined that DUXAP8 displays markedly different expression levels among cancers.

**FIGURE 1:**
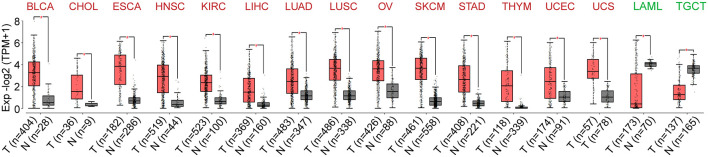
The expression of double homeobox A pseudogene (DUXAP) pattern across pancancer. DUXAP8 expression was dysregulated in several cancer types, and elevated in the bladder urothelial carcinoma (BLCA), cholangiocarcinoma (CHOL), esophageal carcinoma (ESCA), head and neck squamous cell carcinoma (HNSC), kidney renal clear cell carcinoma (KIRC), liver hepatocellular carcinoma (LIHC), lung squamous cell carcinoma (LUSC), ovarian serous cystadenocarcinoma (OV), skin cutaneous melanoma (SKCM), stomach adenocarcinoma (STAD), thymoma (THYM), uterine corpus endometrial carcinoma (UCEC), and uterine carcinosarcoma (UCS). However, the downregulated DUXAP8 expression was observed in the low DUXAP8 TPM in acute myeloid leukemia (LAML) and testicular germ cell tumor (TGCT).

We observed that DUXAP8 produces relatively high transcripts per million (TPM) in bladder urothelial carcinoma (BLCA), cholangiocarcinoma (CHOL), esophageal carcinoma (ESCA), head and neck squamous cell carcinoma (HNSC), kidney renal clear cell carcinoma (KIRC), liver hepatocellular carcinoma (LIHC), lung adenocarcinoma (LUAD), lung squamous cell carcinoma (LUSC), ovarian serous cystadenocarcinoma (OV), skin cutaneous melanoma (SKCM), stomach adenocarcinoma (STAD), thymoma (THYM), uterine corpus endometrial carcinoma (UCEC), and uterine carcinosarcoma (UCS) tissues compared to corresponding normal tissues. We also observed low DUXAP8 TPM in acute myeloid leukemia (LAML) and testicular germ cell tumors (TGCT) compared to normal tissues (Figure 1).

These results indicate that upregulated DUXAP8 expression in tumor tissues compared with adjacent normal tissues might be a useful indicator in cancer diagnosis.

### Long Non-coding RNA Double Homeobox A Pseudogene 8 Displays Potential as a Novel and Broadly Useful Biomarker for Cancer Prognosis

**FIGURE 2:**
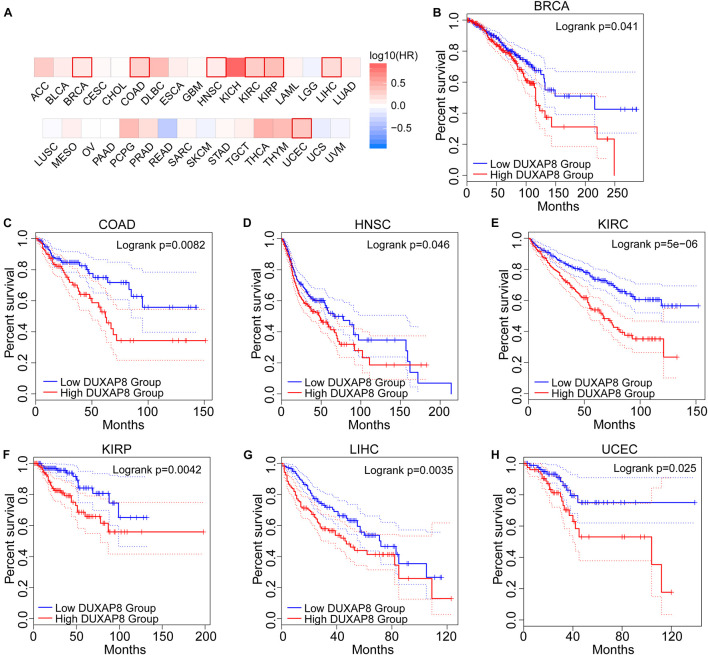
The prognostic prediction of DUXAP pattern in different cancer types. **(A)** DUXAP8 expression correlated with poor overall survival in seven cancers. **(B–H)**, patients with overexpressed DUXAP8 had a shorter survival time than patients with downregulated DUXAP8 expression in breast invasive carcinoma (BRCA) (*p* = 0.041), COAD (*p* = 0.0094), HNSC (*p* = 0.047), KIRC (*p* = 7.6e–6), KIRP (*p* = 0.0057), LIHC (*P* = 0.0038), and UCEC (*p* = 0.03).

To further explore the prognostic role of DUXAP8 expression levels in cancers, we used the GEPIA2 survival analysis module and found that high DUXAP8 mRNA expression correlated with poor overall survival in seven cancers. Specifically, patients with high DUXAP8 expression levels had a shorter survival time than patients with low DUXAP8 expression levels in breast invasive carcinoma (BRCA) (*p* = 0.041), colon adenocarcinoma (COAD) (*p* = 0.0094), HNSC (*p* = 0.047), KIRC (*p* = 7.6e−6), kidney renal papillary cell carcinoma (KIRP) (*p* = 0.0057), LIHC (*P* = 0.0038), and UCEC (*p* = 0.03) (Figure 2). In summary, high DUXAP8 expression was positively associated with shorter survival times and worse prognosis. These results indicate that in most tumor tissues, high DUXAP8 expression has potential as a novel prognostic indicator of cancer progression.

### Associations Between Long Non-coding RNA Double Homeobox A Pseudogene 8 and Clinical Characteristics Based on Current Literature

Double homeobox A pseudogene 8 expression was upregulated in various cancers, such as bladder cancer, hepatocellular carcinoma (HCC), colorectal cancer (CRC), lung cancers, oral cancers, gastric cancer, ovarian cancer, pancreatic cancer, neuroblastoma, and pancreatic cancer. The association between the clinical characteristics and DUXAP8 expression was listed in Table 1.

**TABLE 1:** The clinical information of double homeobox A pseudogene 8 (DUXAP8) in pan-cancers.

Cancer types	Clinical tumor tissues	Expression level	Clinical characteristics	References
Bladder cancer	31 pairs	upregulated	High DUXAP8 expression indicating poor prognosis and advanced tumor stages	Lin et al., 2018
HCC	TCGA database	upregulated	High DUXAP8 expression indicating poor prognosis	Wang et al., 2020
HCC	55 pairs	upregulated	High DUXAP8 expression indicating poor prognosis	Hu et al., 2020
HCC	50 pairs	upregulated	High DUXAP8 expression is associated with larger tumor size, tumor stages, and distant metastasis	Wei et al., 2020
HCC	TCGA database	upregulated	Higher DUXAP8 expression had poor prognosis	Jiang et al., 2019
HCC	TCGA database	upregulated	High DUXAP8 was positively correlated with elder patients (year over 60), advanced stages (stage III/IV), and vascular invasion	Yue et al., 2019
HCC	HCC microarray profiles	upregulated	Higher DUXAP8 was detected in stage II/III HCC samples compared to stage I HCC. High DUXAP8 expression predicted poor OS and correlated with lymph nodes metastasis, and tumor stages	Zhang et al., 2020
CRC	127 pairs	upregulated	High DUXAP8 was correlated with shorter OS	Du et al., 2019
CRC		upregulated	CRC patients in stage I-II presented a lower level of DUXAP8 than those in stage III-IV, and patients with larger tumor size remained higher DUXAP8 expression	Gong et al., 2019
CRC	30CRC patients	upregulated	High DUXAP8 expression indicating poor prognosis	He et al., 2020
RCC	5 public RCC microarray gene profiling datasets	upregulated	High DUXAP8 expression indicating poor prognosis	Xu et al., 2017; Huang et al., 2018; Chen et al., 2019
Lung adenocarcinoma	45 patients	upregulated	High DUXAP8 expression was associated with advanced tumor stages, larger tumor sizes, and metastasis	Liu et al., 2021
Non-small cell lung cancer	43 patients	upregulated	DUXAP8 was upregulated and associated with low overall survival in NSCLC patients	Yang et al., 2019
Non-small cell lung cancer	66 pairs	upregulated	high expression of DUXAP9 is closely associated with advanced tumor stages, larger tumor sizes, lymph node metastasis, and associated with shorter overall survival	Yin et al., 2020
Non-small cell lung cancer	54 patients	upregulated	High expression of DUXAP8 was associated with advanced tumor stages, larger tumor sizes, lymph node metastases, and poor prognosis.	Ji et al., 2020
Non-small cell lung cancer	78 pair of patients	upregulated	Increased DUXAP8 expression was associated with poor prognosis	Sun et al., 2017
Oral cancer	GDC Data Portal and Gene Expression Omnibus (GEO) datasets	upregulated	High expressed DUXAP8 was associated with shorter OS time	Chen et al., 2020b
Gastric cancer	72 pairs	upregulated	High DUXAP8 was associated with advanced tumor stages, larger tumor size, lymphatic metastasis, and poor prognosis	Ma et al., 2017
Ovarian cancer	33 pairs	upregulated	High DUXAP8 expression indicating poor prognosis	Li J.R. et al., 2021
Pancreatic cancer	24 paired	upregulated	DUXAP8 was significantly upregulated in pancreatic cancer tissues	Li J.R. et al., 2021
Pancreatic cancer	58 patients	upregulated	High DUXAP8 expression obtained larger tumor size and TNM stages, and associated with shorter overall survival time	Lian et al., 2018
Neuroblastoma	Gene Expression Omnibus (GEO) database	upregulated	High DUXAP8 was higher in NB tumor tissues in T4 stage than that in T1 stage. High DUXAP8 was associated with poor prognosis	Nie et al., 2020

#### Bladder Cancer

Bladder cancer has become one of the most common cancers worldwide. More than 2 million woman were diagnosed patients were diagnosed, and more than 0.6 million death in 2018 (Wigner et al., 2021). LncRNAs have been identified as novel essential regulators of various human cancers. DUXAP8 is reportedly upregulated in bladder cancer tissues (Jiang et al., 2018; Lin et al., 2018). Researchers have detected substantially elevated lncRNA DUXAP8 expression in bladder cancer tissues compared with adjacent normal tissues (Lin et al., 2018). High DUXAP8 expression is correlated with shorter overall survival time. Advanced stage bladder cancer patients frequently have higher DUXAP8 mRNA expression levels than stage I and stage II patients (Lin et al., 2018).

#### Hepatocellular Carcinoma

Hepatocellular carcinoma is among the most common malignant tumor types, and has a poor prognosis in part due to late diagnosis (Huang et al., 2020). The molecular mechanisms underlying HCC pathogenesis have not been comprehensively elucidated (Huang et al., 2020). Emerging evidence suggests that lncRNAs are widely expressed and might function as promising therapeutic targets and prognostic indicators of various diseases and cancers (Hu et al., 2019; Statello et al., 2021). DUXAP8 expression is markedly upregulated in HCC tumor tissues compared to that in corresponding normal tissues (Yue et al., 2019; Hu et al., 2020; Wang et al., 2020; Wei et al., 2020; Zhang et al., 2020). Jiang et al. (2019) and Wang et al. (2020) found that higher DUXAP8 expression is strongly associated with poor prognosis in HCC (Wang et al., 2020). They also showed that DUXAP8 RNA affects mitotic nuclear division, histone binding, regulation of cell cycle phase transitions, oxidative phosphorylation, cell division, and the tricarboxylic acid cycle, indicating that DUXAP8 can act as an oncogene in HCC progression (Wang et al., 2020). Hu et al. (2020) also showed that DUXAP8 is substantially upregulated in HCC tissues, and found that DUXAP8 was markedly elevated in advanced stage III/IV tumors compared with stage I/II tumors (Hu et al., 2020). Wei et al. (2020) applied GEPIA and found that DUXAP8 was upregulated in HCC tissues. They also correlated upregulated DUXAP8 expression with larger tumor size, more advanced tumor stage, and distant metastases. Patients with higher DUXAP8 expression were associated with shorter overall survival times (Wei et al., 2020). Yue et al. (2019) discovered that DUXAP8 was considerably upregulated in elderly patients (>60 years), tumors in advanced stages (stage III/IV), and during vascular invasion. Similarly, they found upregulated DUXAP8 expression in stage II/III HCC samples relative to stage I HCC samples, and saw an association with poor prognosis (Zhang et al., 2020).

#### Colorectal Cancer

Colorectal cancer is the third most common malignancy. Involvement of a variety of genetic and epigenetic changes has been reported in CRC initiation and progression (Okugawa et al., 2015; Siskova et al., 2020). LncRNAs have been reported to play important roles in epigenetic alterations, revealing their potential as novel targets for CRC prevention and treatment (Chen et al., 2021; Liao et al., 2021). Gong et al. (2019) found that CRC patients in stages I-II presented with lower levels of tumor DUXAP8 than those in stage III-IV, and patients with larger tumor sizes expressed higher levels of DUXAP8. Another study demonstrated increased DUXAP8 expression in CRC tissues compared with paracarcinoma tissues, and showed that high DUXAP8 expression was indicative of shorter overall survival time (He et al., 2020). These data suggest the carcinogenic potential of DUXAP8 in CRC.

#### Renal Cell Carcinoma

Renal cell carcinoma remains one of the most lethal urological malignancies (Capitanio et al., 2019). Epigenetic modifications are common in RCC, suggesting that these modifications play an important role in RCC initiation and progression (Joosten et al., 2018). Researchers have discovered that many lncRNAs are upregulated and associated with poor prognosis in RCC (Zhai et al., 2017). Many researchers have investigated public RNA sequencing data and microarray gene profiling data from RCC patients, and found that DUXAP8 was markedly upregulated in RCC tumor tissues compared with adjacent para-tumor tissues (Xu et al., 2017; Huang et al., 2018; Chen et al., 2019). They have also shown that increased DUXAP8 expression correlates with poor prognosis in RCC (Xu et al., 2017; Huang et al., 2018; Chen et al., 2019).

#### Lung Cancer

Lung cancer is the leading cause of cancer-related deaths worldwide (Loewen et al., 2014). LncRNAs are a new class of cancer regulators that govern fundamental biochemical and cellular processes in lung cancer (Feng et al., 2019). DUXAP8 expression is substantially increased in LUAD tumor tissues. High DUXAP8 expression is closely associated with advanced tumor stages, larger tumor sizes, and metastasis (Yang et al., 2019; Yin et al., 2020; Liu et al., 2021). Ji et al. (2020) have also revealed that DUXAP8 is notably increased in non-small-cell lung cancer (NSCLC) tissues, and is associated with lymph node metastases and advanced tumor stages. Sun et al. (2017) analyzed tumor tissue and normal tissue from 78 pairs of patients, and found that DUXAP8 was notably increased in tumor tissues compared with normal tissues. Elevated DUXAP8 expression has been positively correlated with tumor size, lymph node metastasis, tumor stage, shorter survival time, and shorter progression-free survival time (Sun et al., 2017; Li L.M. et al., 2021).

#### Ovarian Cancer

Double homeobox A pseudogene 8 is markedly upregulated in ovarian cancer, where elevated expression is associated with shorter overall survival time (Lian et al., 2018; Li J.R. et al., 2021).

#### Oral, Esophageal, Gastric, and Colon Cancers

Digestive tract cancers are a group of malignant cancers that together represent the most common cause of cancer-related deaths worldwide (Lai et al., 2019; Stokłosa et al., 2020). DUXAP8 is substantially upregulated in oral cancer tissues compared to normal tissues (Chen et al., 2020b). Increased DUXAP8 expression is negatively associated with overall patient survival time (Chen et al., 2020b). In esophageal cancer, DUXAP8 expression level is closely related to clinical stage, lymph node metastasis, and overall survival (Liu et al., 2018; Xu et al., 2018). Increased DUXAP8 expression has also been detected in gastric cancer tissues compared to corresponding normal tissues (Ma et al., 2017). In colon cancer research, elevated DUXAP8 expression has been positively correlated with advanced stages, lymph node metastasis, and shorter overall survival time (Ma et al., 2017).

#### Double Homeobox A Pseudogene 8 in Ovarian and Pancreatic Cancers

Double homeobox A pseudogene 8 expression was markedly upregulated in tumor tissues compared to corresponding adjacent pancreatic tissue samples (Lian et al., 2018; Li J.R. et al., 2021). Increased DUXAP8 expression was also found to closely associate with larger tumor size, advanced stage, and shorter overall survival time (Lian et al., 2018).

#### Double Homeobox A Pseudogene 8 in Other Cancers

Studies have also revealed that DUXAP8 is substantially upregulated in neuroblastoma and papillary thyroid carcinoma tissues compared to corresponding adjacent normal tissues (Nie et al., 2020). Levels of DUXAP8 detected in neuroblastoma tumor tissues have been higher in T4 stage than in T1 stage, and elevated DUXAP8 expression is associated with worse prognosis (Nie et al., 2020).

### Double Homeobox A Pseudogene 8 Regulatory Mechanism in Cancer Initiation and Tumor Progression

Double Homeobox A Pseudogene 8 is markedly upregulated in various cancer tissues, which plays an important role in cancer initiation and progression. In this review, we comprehensively summarize existing research on DUXAP8 functional roles in various cancers, such as bladder cancer, HCC, CRC, RCC, NSCLC, esophageal cancer, oral cancer, gastric cancer, neuroblastoma, thyroid carcinoma, and breast cancer.

**FIGURE 3:**
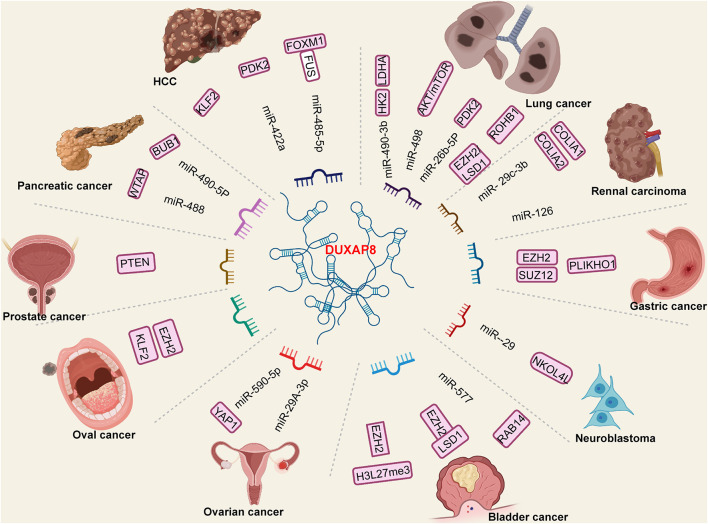
The specific long non-coding RNA (lncRNA)-miRNA oncogene regulation mechanism of DUXAP8 in various cancers. The DUXAP8 was significantly overexpressed in tumor tissues. The increased DUXAP8 function as an oncogene and a sponge which targeting mi-RNAs and activated downstream oncogene pathways.

A mechanism of regulation is illustrated in Figure 3. In bladder, Lin et al. (2018) have demonstrated that DUXAP8 downregulated phosphatase level and facilitated tumor cell progression. Jiang et al. (2018) have demonstrated that DUXAP8 knockdown in bladder cancer cells can inhibit tumor cell invasion and induce tumor cell apoptosis. In a study by Hu et al. (2020), DUXAP8 acted as an oncogene when expressed at elevated levels, promoting and maintaining multiple malignant phenotypes by sequestering miR-485-5p to regulate the DUXAP8/Forkhead box M1 axis. Wei et al. (2020) demonstrated that DUXAP8 could sequester miR-422a, thus enhancing pyruvate dehydrogenase kinase isozyme 2 (PDK2) expression in HCC cell lines and promoting HCC malignant phenotypes. Yue et al. (2019) also demonstrated that DUXAP8 knockdown substantially inhibited the proliferation, migration, and invasion abilities of HCC cell lines. DUXAP8 can also sequester miR-490-5p, activating budding uninhibited by benzimidazole-1 (BUB1) expression and facilitating tumor proliferation and invasion (Zhang et al., 2020). Increased DUXAP8 expression allows it to sequester miR-577, enhancing the levels of ras-related protein 14 and promoting tumor proliferation and invasion (Du et al., 2019). Gong et al. (2019) demonstrated that DUXAP8 knockdown may suppress the proliferative, migratory, and invasive abilities of CRC cells. Specifically, in an *in vitro* study, increased DUXAP8 apparently potentiated the expression of lysine-specific histone demethylase 1A (LSD1) and Enhancer of zeste homolog 2 (EZH2), thereby accelerating CRC cell malignant activities. He et al. (2020) reported that increased DUXAP8 expression activates CRC cell proliferation and inhibits apoptosis, and that DUXAP8 interacts with EZH2 and H3K27me3. These studies suggest that DUXAP8 displays potential as a novel therapeutic target for CRC. Reports indicate that in RCC, DUXAP8 pseudogenes promote tumor growth *via* suppression of the miR-29c-3p, collagen type I alpha 1 (COL1A1)/COL1A2 axis in RCC (Chen et al., 2019). Xu et al. (2017) have shown that DUXAP8 knockdown markedly inhibited RCC cell invasion abilities. Huang et al. (2018) have demonstrated that increased DUXAP8 lncRNA might promote RCC cell proliferation and invasion by regulating the miR-126/cell death abnormal-axis. In lung cancer, increased DUXAP8 promotes cancer proliferation and suppresses apoptosis by targeting miR-26b-5p (Liu et al., 2021). Yang et al. (2019) found that DUXAP8 knockout substantially inhibited cell invasion, whereas DUXAP8 overexpression promoted cell invasion. Another study demonstrated that increased DUXAP8 expression might promote lung cancer cell growth, metastasis, and glycolysis. Mechanistically, increased DUXAP8 expression inhibited miR-409-3p expression to upregulate HK2 and LDHA expression (Yin et al., 2020). In NSCLC, increased DUXAP8 could inhibit tumor growth and metastasis through reciprocal regulation of miR-498 and TRIM44 *in vivo* (Ji et al., 2020). DUXAP8 acts as an oncogene, facilitating tumor cell proliferation, migration, and invasion by interacting with EZH2 and LSD1 to suppress their activities (Sun et al., 2017). In oral cancer, increased DUXAP8 promotes tumor proliferation, migration, and invasion by activating the EZH2/Kruppel-like factor 2 (KLF2) axis (Chen et al., 2020b). In esophageal cancer, DUXAP8 knockdown may inhibit tumor proliferation, migration, and invasion (Liu et al., 2018; Xu et al., 2018). In gastric cancer regulatory mechanisms, increased DUXAP8 expression promotes tumor cell proliferation and tumorigenesis, partly through epigenetic silencing of pleckstrin homology domain-containing family O member 1 transcription by binding to polycomb-repressive complex 2 (Ma et al., 2017). Marked upregulation of DUXAP8 in colon cancer tissues compared with peritumor tissues has also been validated, and has been associated with tumor stage (III/IV) and larger tumor sizes (Chen et al., 2020a). In colon cancer cells, DUXAP8 accelerated malignant progression by targeting LSD1 and EZH2 (Chen et al., 2020a). Meng et al. (2020) demonstrated that DUXAP8 is involved in ovarian cancer proliferation, migration, and invasion. Increased DUXAP8 promotes yes-associated protein 1 (YAP1) expression by inhibiting miR-590-5p expression in ovarian cancer cells (Meng et al., 2020). In pancreatic cancer, DUXAP8 promotes the migration and invasion of pancreatic cancer cells by sequestering miR-448 and focal adhesion kinase (Li J.R. et al., 2021). Another study demonstrated that DUXAP8 regulates pancreatic cancer cell proliferation, migration, and invasion through epigenetic silencing of CDKN1A and KLF2 expression (Lian et al., 2018). Studies have also revealed that DUXAP8 exacerbates the malignancy of neuroblastoma cells *via* a miR-29/nucleolar protein 4 like axis *in vivo* (Nie et al., 2020). In papillary thyroid carcinoma, DUXAP8 binding of miR-223-3p upregulates CXC chemokine receptor 4 signaling (Pang and Yang, 2021). Specifically, increased DUXAP8 expression was positively associated with SOS1, c-Myc, and CCND1 expression. DUXAP8 is also markedly upregulated in breast cancer. Increased oncogenic DUXAP8 function in sequestering miR-29a-3b enhances oncogene suppressor APC domain containing 2 expression and its corresponding oncogenic signaling pathways (Yang et al., 2021). These studies may provide novel insights into the etiology of cancers, and valuable lncRNA candidates for further investigation of lncRNA roles in cancer progression.

## Conclusion and Further Perspectives

Expression of the novel lncRNA DUXAP8 has been described in various cancers. DUXAP8 levels are substantially upregulated in tumor tissues compared with adjacent normal tissues. High DUXAP8 expression correlates with shorter overall survival time and worse prognosis. Higher DUXAP8 levels indicate larger tumor sizes and advanced tumor stages. Aberrant DUXAP8 expression is closely related to many clinicopathological parameters. Therefore, in clinical applications, DUXAP8 displays potential as a novel indicator for the early diagnosis and prediction of tumor progression and outcome. However, DUXAP8 mRNA expression has not been fully characterized in blood and other biological samples. With the development of advanced technologies, the expression landscape of DUXAP8 in patient body fluids will soon be described. DUXAP8 is also involved in multiple mechanisms regulating cancer initiation and progression, indicating strong potential for DUXAP8 as a therapeutic target. In conclusion, DUXAP8 is a promising cancer indicator and therapeutic target for cancer.

## Author Contributions

CX and JJ designed the study. CX drafted the manuscript. XC and JJ revised the manuscript. All authors read and approved the final manuscript.

## Conflict of Interest

The authors declare that the research was conducted in the absence of any commercial or financial relationships that could be construed as a potential conflict of interest.

## Publisher’s Note

All claims expressed in this article are solely those of the authors and do not necessarily represent those of their affiliated organizations, or those of the publisher, the editors and the reviewers. Any product that may be evaluated in this article, or claim that may be made by its manufacturer, is not guaranteed or endorsed by the publisher.
